# Supplementary material for: Placenta-associated adverse pregnancy outcomes in women experiencing mild or severe hyperemesis gravidarum – a systematic review and meta-analysis
Source: BMC Pregnancy Childbirth. 2023 May 24;23:375. doi: 10.1186/s12884-023-05691-6 (PMC10207696; doi:10.1186/s12884-023-05691-6)
Supplement: Supplementary file 2 — Additional file 2. [file 12884_2023_5691_MOESM2_ESM.pdf]

Secondary outcomes

Small for gestational age (SGA) was reported in 12 studies with a total number of 7,797,344 participants. Seven studies reported SGA in women with HG and were pooled in a meta-analysis, which resulted in OR 1.24, 95% CI 1.13 to 1.35, (Figure S1). Due to high heterogeneity, the studies on women with NVP could not be pooled in a meta-analysis. Three of four studies showed an increased risk for SGA in women with NVP, though only two significant. The fourth study showed a significant decreased risk for SGA in women with NVP.

	Women with HG		Women without HG			Odds Ratio
Study or Subgroup	Events	Total	Events	Total	Weight	M-H, Random, 95% CI
Bolin 2013	381	12270	26683	1133872	24.0%	1.33 [1.20, 1.47]
Dodds 2006	137	1270	15217	154821	14.4%	1.11 [0.93, 1.33]
Fejzo 2013	7	254	3	308	0.4%	2.88 [0.74, 11.26]
Fiaschi 2018	7662	66797	393594	4388115	35.1%	1.31 [1.28, 1.35]
Koudijs 2016	60	307	212	1277	6.2%	1.22 [0.89, 1.68]
Kuru 2012	5	72	12	89	0.6%	0.48 [0.16, 1.43]
Roseboom 2011	237	2190	117309	1197028	19.3%	1.12 [0.98, 1.28]
**Total (95% CI)**		**83160**		**6875510**	**100.0%**	**1.24 [1.13, 1.35]**
Total events	8489		553030			

Heterogeneity: $Tau^2 = 0.01$; $Chi^2 = 13.52$, df = 6 (P = 0.04); $I^2 = 56\%$

Test for overall effect: Z = 4.80 (P < 0.00001)

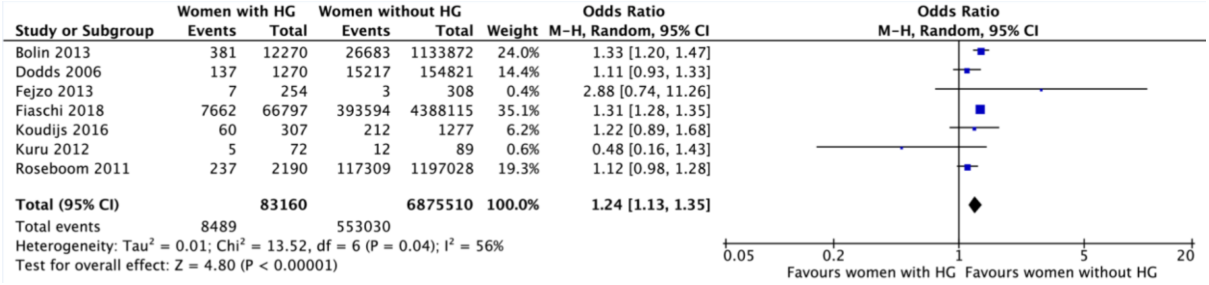

Figure S1. Meta-analysis for small for gestational age (SGA).

Forest plot showing the meta-analysis for SGA in women with HG. Odds ratios with 95% confidence intervals (CI) are reported. The heterogeneity is showed as the value of I^2.

Low birth weight (LBW) was reported in 11 studies with a total number of 7,824,780 participants. The studies reporting LBW in women with HG were pooled in a meta-analysis, which resulted in OR 1.35, 95% CI 1.26 to 1.44, (Figure S2). Due to high heterogeneity, the studies on women with NVP could not be pooled in a meta-analysis. Three of these five studies showed a significant reduced risk for LBW in women with NVP. The other two studies showed an increased risk for LBW in women without NVP, although only one was statistically significant. The study by Weigel *et al.* (34) was excluded due to missing data.

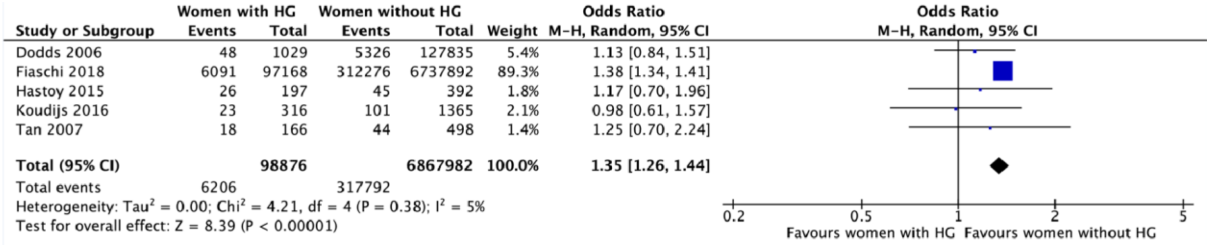

Figure S2. Meta-analysis for low birth weight (LBW).

Forest plot showing the meta-analysis for LBW in women with HG. Odds ratios with 95% confidence intervals (CI) are reported. The heterogeneity is showed as the value of I^2.

Fetal sex was reported in eight studies with a total number of 1,446,151 participants. The studies reporting women with HG were pooled in a meta-analysis, which resulted in OR 1.36, 95% CI 1.15 to 1.60, (Figure S3). Due to high heterogeneity, the studies on women with NVP could not be pooled in a meta-analysis. Three of four studies showed higher probability for the fetus being female in women with NVP. The other study showed a significant lower probability of the fetus being female in women with NVP, contributing to over half of the total number of participants.

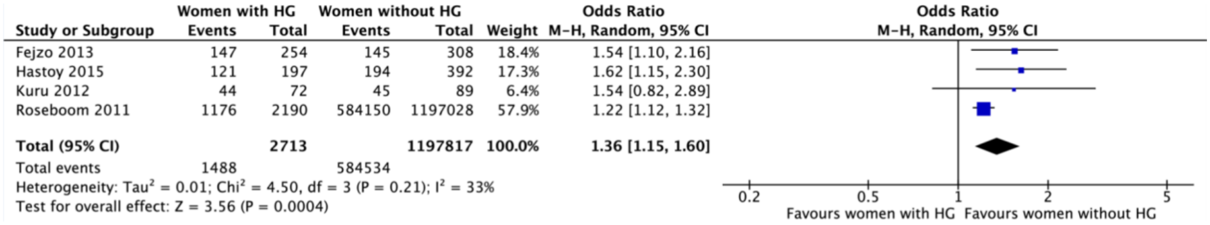

Figure S3. Meta-analysis for fetal sex (female).

Forest plot showing the meta-analysis for fetal sex (female) in women with HG. Odds ratios with 95% confidence intervals (CI) are reported. The heterogeneity is showed as the value of I^2.

Intrauterine fetal death (IUFD) was reported in seven studies with a total number of 10,650,249 participants. A meta-analysis was performed for the six studies reporting IUFD in women with HG, which resulted in OR 0.94, 95% CI 0.86 to 1.02, (Figure S4). The study by

Fiaschi *et al.* (11) reported this outcome in two different groups: one group with only fetal deaths and one group where one fetus died, and one lived (multiple pregnancy). In the meta-analysis the groups were analysed together. There was only one study reporting IUFD in women with NVP, indicating a higher risk for IUFD for women with NVP, but the results were not significant.

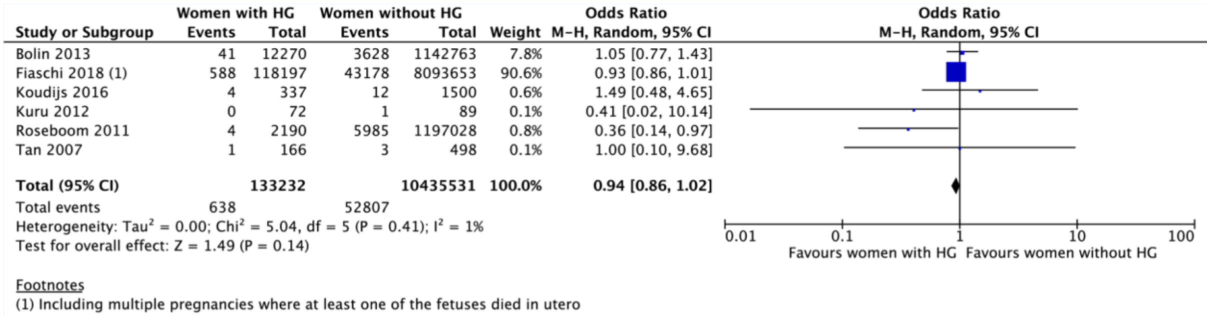

Figure S4. Meta-analysis for intrauterine fetal death (IUFD).

Forest plot showing the meta-analysis for IUFD in women with HG. Odds ratios with 95% confidence intervals (CI) are reported. The heterogeneity is showed as the value of I^2.

Congenital malformations were reported in four studies with a total of 704,208 participants. A meta-analysis was not performed due to high heterogeneity. Two of the studies showed a decreased risk for congenital malformations in women with NVP and two studies showed an increased risk for women with NVP or HG.

Placental abruption was reported in two studies with a total of 9,367,900 participants. A meta-analysis was not performed since the outcome was only reported in two studies. Both studies showed significant results of an increased risk for placental abruption in women with HG.

Rate of multiple pregnancies was reported by two studies with a total of 666,144 participants. A meta-analysis was not performed since the outcome was only reported in two studies. Both studies showed higher rates of multiple pregnancies in women with NVP or HG.

Need for admission to NICU was reported in two studies with a total of 9,411,068 participants. A meta-analysis was not performed since the outcome was only reported in two studies. Both studies showed higher rates of admission to NICU in women with HG.
